# Microbiome Composition and Its Impact on the Development of Allergic Diseases

**DOI:** 10.3389/fimmu.2020.00700

**Published:** 2020-04-23

**Authors:** Diego G. Peroni, Giulia Nuzzi, Irene Trambusti, Maria Elisa Di Cicco, Pasquale Comberiati

**Affiliations:** ^1^Department of Clinical and Experimental Medicine, Section of Pediatrics, University of Pisa, Pisa, Italy; ^2^Department of Clinical Immunology and Allergology, I.M. Sechenov First Moscow State Medical University, Moscow, Russia

**Keywords:** allergy, asthma, atopic dermatitis, food allergy, health outcomes, immune system, children, microbiome

## Abstract

Allergic diseases, such as food allergy (FA), atopic dermatitis (AD), and asthma, are heterogeneous inflammatory immune-mediated disorders that currently constitute a public health issue in many developed countries worldwide. The significant increase in the prevalence of allergic diseases reported over the last few years has closely paralleled substantial environmental changes both on a macro and micro scale, which have led to reduced microbial exposure in early life and perturbation of the human microbiome composition. Increasing evidence shows that early life interactions between the human microbiome and the immune cells play a pivotal role in the development of the immune system. Therefore, the process of early colonization by a “healthy” microbiome is emerging as a key determinant of life-long health. In stark contrast, the perturbation of such a process, which results in changes in the host-microbiome biodiversity and metabolic activities, has been associated with greater susceptibility to immune-mediated disorders later in life, including allergic diseases. Here, we outline recent findings on the potential contribution of the microbiome in the gastrointestinal tract, skin, and airways to the development of FA, AD, and asthma. Furthermore, we address how the modulation of the microbiome composition in these different body districts could be a potential strategy for the prevention and treatment of allergic diseases.

## Introduction

Over the last few decades, many developed and fast-growing countries worldwide have registered a dramatic increase in the prevalence of allergic diseases, such as asthma, AD, and FA, which currently pose a substantial burden to healthcare systems (1, 2). Thus far, the genetic and environmental drivers of the rapid rise in allergy prevalence remain to be more fully elucidated.

Notably, the evolution of the allergy epidemic has closely paralleled radical environmental and lifestyle changes, such as progressive industrialization and urbanization, widespread sanitation programs and antibiotics use, physical inactivity and highly processed diets. All these changes have led to reduced microbial exposure in early life and loss of microbial biodiversity (3).

Accumulating evidence points to a central role of the human microbiome perturbation in the rising prevalence of allergic diseases. The human microbiome comprises bacteria, viruses, fungi, protozoans, and archaea, which colonize primarily the gastrointestinal tract, but also the airways and the skin surface from the first days of life and gradually develop and diversify concomitantly with the physiological growth of the individual. The resident microbial communities in the human gut and other organs have been shown to modulate both the innate and acquired immune responses. Recent data show that several environmental drivers can affect the microbiome colonization, composition and metabolic activity in infancy, and alter the host functions for nutrition and immunity (4). Indeed, the process of early colonization by a “healthy” microbiome is emerging as a key determinant of life-long health, whereas the perturbation of such a process, has been associated with greater susceptibility to immune-mediated disorders later in life, including allergic diseases (5).

The recent introduction of the next-generation sequencing and genomic analysis to identify different microbial species has led to a greater knowledge of the complex role of the human microbiome in the pathogenesis of FA, AD, and asthma. Here, we review recent findings on the potential role of the human microbiome in the gastrointestinal tract, the skin, and the airways to the development of allergic diseases, and we address how the modulation of the microbiome composition could be a potential therapeutic or even preventive strategy for such disorders.

## Early Life Factors Modulating Gut Microbiome Composition

**FIGURE 1:**
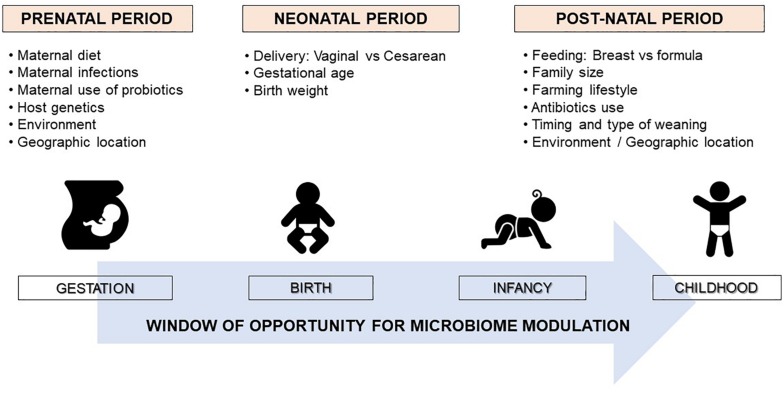
Factors shaping the human microbiome development. The neonatal microbiome is a delicate and highly dynamic ecosystem that undergoes rapid changes in composition in the first few years of life determined by several pre and perinatal factors. The maturation of the gut microbiota toward an adult-like structure largely occurs by the age of 2 or 3 years. Therefore, early infancy could be a critical period for modulating the microbiota to promote healthy growth and development.

It is well established that microbiome composition changes dynamically in the first few years of life and can be influenced by several prenatal and postnatal environmental and host-related factors (Figure 1) (6). Among these factors, mounting evidence shows that some perinatal factors, such as mode of delivery, breastfeeding, early antibiotic use, and timing and type of complementary feeding, can significantly modulate the gut microbiome composition, which is emerging as a key determinant in developing immune tolerance responses to different antigens (7). The gut microbiome of newborns delivered by cesarean section shows a lower level of commensal bacteria typically found in those born vaginally and high concentrations of opportunistic pathogens typically found in the hospital environment, such as *Enterococcus*, *Enterobacter*, and *Klebsiella* species (8). These differences largely even by the time babies are weaned around 6 to 9 months, except for commensal bacteria *Bacteroides*, which remain absent or at very low levels in most cesarean section infants. Of note, the effect of the cesarean section on the infant microbiome seems to be related to maternal antibiotic exposure before the delivery (8).

Breast milk contributes to the development of healthy gut microbiome. BM contains essential micronutrients and prebiotic compounds, which support the colonization and growth of commensal bacteria, and several immune active factors, oligosaccharides and microbes, which could all modulate host immune responses (9). Term infants born vaginally and breastfed exclusively seem to have the most “beneficial” gut microbiome, with the highest concentration of ***Bifidobacteria*** and lowest numbers of ***Clostridium difficile*** and ***Escherichia coli*** (10).

Shifting from exclusively breastfeeding to complementary feeding at weaning increases the prevalence of *Bacteroides, Bilophila, Roseburia, Clostridium, and Anaerostipes*, and progressively leads to the establishment of an adult-type microbiome (11). In particular, the introduction of solid foods modulates gut microbiome shifting from *Bifidobacterium*-dominant to *Bacteroidetes*- and *Firmicutes*-dominant species, such as the *Clostridium coccoides* and *Clostridium leptum* groups (12). The introduction of solid foods also induces a sustained increase in fecal SCFA levels and expression of genes associated with the adult microbiome’s core metabolic functions, such as polysaccharide breakdown, vitamin biosynthesis, and xenobiotic degradation (13).

The first 1000 days of life (i.e., the period from conception to age 2 years) seem to represent the critical window of opportunity for microbiome modulation (Figure 1) (6, 14). After this period, the gut microbiome tends to acquire an adult-like configuration with distinct microbial community composition and functions (15). However, several factors can induce significant perturbations to the gut microbiome composition later in life, such as long-term dietary changes, or frequent or prolonged use of antibiotics (13, 16). Notably, a very recent multi-omics integrative analysis showed that antibiotic use in adults induced alterations to the gut microbiome which adversely affected immunogenicity and responses to influenza vaccination (17).

## How the Gut Microbiome Can Influence Immune Responses

Neonatal and infant gut microbiome appear to be involved in gut tolerance modulation and immune system “education” (18, 19). Germ-free animal experiments best describe this mutualistic relationship in animals (20–27). These data may support such a relationship in humans.

Indeed, some recent human studies have addressed the role of the gut microbiome on adaptive and innate immunity in the context of allergic diseases. Christmann et al. (28), reported lower IgG responses to specific clusters of microbiota antigens in infants who then developed allergic disorders in childhood (including skin, respiratory, and food allergies) compared to healthy children. West et al. (29), studied infants at high risk of atopic diseases and showed that depletion of *Proteobacteria* in early infancy is associated with increased Toll-like receptors (TLR)-4 induced innate inflammatory responses, whereas depletion of *Ruminococcaceae* is associated with increased TLR-2 induced innate inflammatory responses. Fujimura et al. (30), reported that infants at risk of asthma have a gut microbial signature with reduced abundance of certain bacteria taxa, such as *Faecalibacterium* and *Bifidobacterium*. Stimulation of adult PBMC with sterile fecal water from these infants then led to increases in CD4 + IL-4 producing cells and reduced regulatory Foxp3 cells. Similarly, Sjödin et al. (31), found that the gut symbiont *Faecalibacterium* correlated with the expression levels of regulatory cytokines in children with multiple allergies, suggesting an opportunity to expand such taxa to promote a regulatory tolerogenic immune response.

## Role of the Microbiome in the Development of Allergic Diseases

The composition of the microbiome varies across different body sites, which constitute unique habitats resulting in varied microbial communities within and between subjects. The greatest concentration and diversity of microorganisms are found in the gastrointestinal tract, which is dominated by facultative and strictly anaerobic bacteria of the phyla *Firmicutes, Bacteroidetes, Actinobacteria, Verrucomicrobia*, and *Proteobacteria* (32).

**FIGURE 2:**
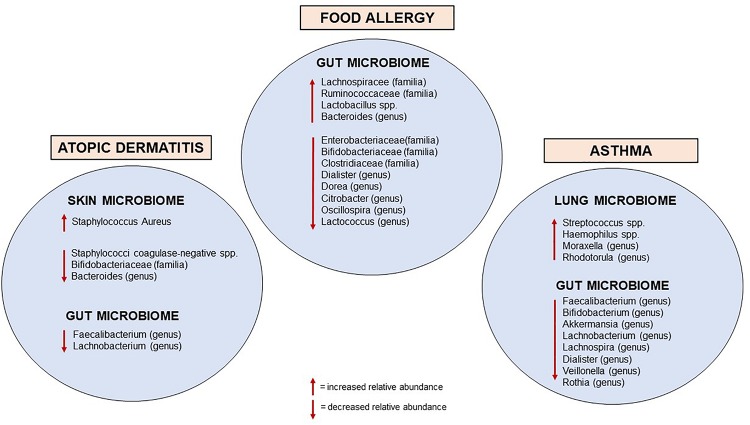
Currently known microbiome perturbations in infancy associated with allergic diseases.

The mechanisms that mediate host-microbe communications are highly complex; a disrupted dialogue due to altered microbiome seems to negatively impact the immune homeostatic networks and may contribute to the development of hypersensitivity reactions to environmental allergens (33). This connection emerged over the last few decades with the proposed “hygiene hypothesis,” based on the epidemiological evidence that environmental drivers increasing early life microbial exposure (such as vaginal delivery, farming life, and furry animals exposure during childhood, large family size, unpasteurized milk consumption and absence of early antibiotic exposure) were associated with a lower risk of developing allergic disorders (34–38). Recent experimental and human investigations have strengthened the mechanistic substance to the hygiene hypothesis, providing evidence for the causal relationship between early life microbial perturbation in the gut, skin, and airways and the development of allergic diseases (Figure 2).

### Microbiome and Food Allergy

The composition and metabolic activities of the gut microbiome seem to be closely linked with the development of oral tolerance (39). Mortha et al. (40), showed that commensal microorganisms favor the crosstalk between innate myeloid and lymphoid cells that contributes to immune homeostasis in the gut and the development of oral tolerance to dietary antigens.

Infants with CMA have more total bacteria, in particular the anaerobic type, compared to healthy controls after 6 months of milk formula assumption. In addition, higher concentrations of *Lactobacilli* and lower concentrations of *Enterobacteria* and *Bifidobacteria* were observed in infants with CMA (41). Bunyavanich et al. showed that *Clostridia* and *Firmicutes* rates were particularly elevated in the gut microbiota of infants whose CMA resolved by 8 years of age (42). Fazlollahi et al. found that the gut microbiome of children with egg allergy had a greater abundance of the genera from *Lachnospiraceae* and *Ruminococcaceae* than those of healthy controls (43). A prospective study comprising 14 children with FA and 87 children with food allergens sensitization, showed that *Haemophilus, Dialister, Dorea*, and *Clostridium* genera were reduced in participants with food sensitization, whereas, the genera *Citrobacter, Oscillospira, Lactococcus*, and *Dorea* were under-represented in participants with FA (44). Furthermore, in subjects with peanut or tree nut allergy, decreased microbial richness and increased concentration of *Bacteroides* species were reported compared to non-allergic controls (45).

Studies in animal models showed that germ-free mice were protected from developing anaphylaxis to cow’s milk if colonized with gut microbiome from healthy infants, but not from infants with CMA (46). The transfer of specific bacterial strains, such as *Bifidobacterium* or *Clostridium* species to mice was shown to reducing the risk of food sensitization, by the induction of mucosal Treg (47). *Clostridia* can also stimulate innate lymphoid cells to produce IL-22, which contributes to strengthen the epithelial barrier and decrease the permeability of the gut to dietary proteins (48). Some functional effects of *Clostridia* in FA likely also occur through their fermentation metabolites, such as butyrate, a SCFA with known immunoregulatory and tolerogenic proprieties (49).

Experimental findings showing that the gut microbiome contributes to the development of food tolerance suggest that microbial modulation could be a potential therapeutic strategy for FA. Although the supplementation of an extensively hydrolyzed milk formula with *Lactobacillus casei* and *Bifidobacterium lactis* did not prove to accelerate the resolution of CMA (50), the administration of extensively hydrolyzed casein formula containing the probiotic *Lactobacillus rhamnosus GG* has been shown to promote CMA resolution at 12, 24, and 36 months, compared to non-supplemented hypoallergenic milk formula (51). Of note, the use of such *Lactobacillus rhamnosus GG*-supplemented formula significantly expanded butyrate-producing bacterial strains in the infant gut microbiome compared to non-supplemented formula (49). In another study, the use of an amino-acid based formula containing a specific synbiotics (i.e., a combination of prebiotic blend of fructo-oligosaccharides and the probiotic strain *Bifidobacterium breve* M-16V) has been shown to modulate the gut microbiome and its metabolic activities also in infants with non-IgE mediated CMA (52–54). Recently, an uncontrolled study suggested that oral supplementation with *Lactobacillus rhamnosus GG* could enhance the efficacy of oral immunotherapy in inducing peanut tolerance and immune changes in children with peanut allergy (55). However, further studies including a control group are required to determine whether modulation of the microbiome during immunotherapy will favor the acquisition of sustained unresponsiveness to food allergens.

### Microbiome and Atopic Dermatitis

Several factors, such as age, gender, ethnicity, climate, ultraviolet exposure, and lifestyle drivers, can influence the composition of skin microbiome (56). The healthy skin microbiome is represented by *Propionibacterium species*, which are mainly found in sebaceous sites, and *Corynebacterium* and *Staphylococcus* species, which are more abundant in moist microenvironments. *Malassezia* is the predominant fungal flora on human skin (56, 57).

Atopic dermatitis is a complex skin disease characterized by epidermal barrier dysfunction, altered innate/adaptive immune responses and impaired skin microbial biodiversity (58). Loss of microbial diversity, with the predominance of the *Staphylococcus aureus* over *Staphylococcus epidermidis*, is a characteristic feature at both acute and chronic skin sites of AD (59), which correlates with AD severity and the risk of allergic sensitization to common allergens (60). *Staphylococcus aureus* contributes to the epidermal barrier disruption through different pathways, including the downregulation of terminal differentiation of epidermal proteins, such as filaggrin and loricrin, and the promotion of the skin proteases activities, which directly damage the skin barrier (61, 62).

Coagulase-negative *Staphylococci*, which include *S. epidermidis, S. hominis* and *S. lugdunensis*, can secrete antimicrobial metabolites that limit *S. aureus* overgrowth and biofilm formation (61). In addition, *S. epidermidis* can also activate TLR2 signaling, which can induce the production of keratinocyte-derived antimicrobial peptides and increase the expession of epidermal tight junction proteins (63). Neonatal colonization of the skin by *S. epidermidis* is associated with the induction of specific Tregs that modulate local activation of host immune responses (64). Indeed, it has been recently shown that skin commensal *Staphylococci* species are significantly reduced at 2 months in infants who later developed AD at 1 year, suggesting that targeted topical modulation favoring early colonization with this genus might reduce the risk of later occurrence of AD (65). These findings, together with evidence that regular application of moisturizers repairs the skin barrier and restores commensal bacterial diversity (66–68), constituted the rationale for ongoing research on the application of topical probiotics, such as *Vitreoscilla filiformis* lysate and *Roseomonas mucosa*, as a potential strategy to modulate the skin microbiome and treat AD (69, 70). Preliminary data also showed that the autologous skin transplantation of antimicrobial coagulase-negative *Staphylococci* strains to human subjects with AD could decrease *S. aureus* overgrowth and colonization (71).

Changes in the gut microbiome seem also to contribute to the development of AD. Patients with AD have lower concentrations of *Bifidobacterium* in the gut microbiome than healthy controls, and these counts are inversely related to the severity of the disease (72). Early gut colonization with *Clostridium difficile* was related to the occurrence of AD (73), and lower *Bacteroidetes* diversity at 1 month was associated with AD at 2 years of age (74). There is evidence that pre- and post-natal supplementation with oral *Lactobacillus* and *Bifidobacterium* strains could reduce the risk of AD in infants due to changes in T cell-mediated responses (75). Finally, a recent large prospective study of gut microbiota showed that *Lachnobacterium* and *Faecalibacterium* were significantly less abundant in those children who developed AD by school-age compared to healthy controls. Notably, the differential abundance of these bacterial taxa was documented throughout infancy, which supports the likelihood of their protective role in the development of AD (76).

### Microbiome and Asthma in Childhood

Accumulating evidence shows that the composition of the lung microbiome in early life can affect the development of respiratory health or disease (77, 78). Preclinical models support a protective role of bacteria against allergic airway inflammation (79, 80).

The phylum *Bacteroides*, particularly *Prevotella* spp., predominate in the lung microbiome of healthy subjects (81, 82). During the first 2 weeks of life, the lung microbiome promotes the transient expression of programmed death-ligand 1 (PDL1) in dendritic cells, which is necessary for the Treg-mediated attenuation of allergic airway responses (83). Epidemiological evidence shows that children who grow up in a farming environment and are exposed to diverse microbial communities since early life have a lower incidence of allergies (84). Notably, the airway colonization by *Streptococcus, Moraxella*, or *Haemophilus* within the first 2 months of life has been associated with the severity of lower respiratory viral infection in the first year of life, and the risk of asthma development later in life (85). The phylum *Proteobacteria* has also been associated with asthma and neutrophilic exacerbations, whereas *Bacteroidetes* with eosinophilic exacerbations, leading to the consideration that distinct mediators and microbiome profiles may represent different clusters of biological exacerbations (86, 87).

Emerging evidence shows that gut microbial perturbations in early life can also influence the development of allergic airway inflammation. Antibiotic use in neonatal mice favors variations in the microbiome composition, which have been associated with alterations in intestinal Tregs and increased susceptibility to airway hyper-responsiveness (88). Similarly, pre- and post-natal exposures to antibiotics in humans have been associated with an increased risk of developing asthma (89). In a recent longitudinal study, Galazzo et al. (76), showed that the bacterial genera *Lachnobacterium*, *Lachnospira* and *Dialister* were significantly decreased in the gut microbiome of infants who developed asthma by school-age compared to healthy controls. Analysis of the gut microbiome at 3 months of age within the Canadian Healthy Infant Longitudinal Development Study (CHILD) showed a reduction in bacterial taxa of the genera *Lachnospira, Veillonella, Faecalibacterium*, and *Rothia* among infants at risk of childhood asthma (90). In another recent observational cohort study, a reduction of *Lachnospiraceae*, *Faecalibacterium*, and *Dialister* at 1 year of age was associated with an increased risk of asthma at 5 years of age (91).

The protective effect of these bacterial taxa on asthma occurrence could be mediated by their fermentation products (92, 93). *Faecalibacterium prausnitzii* ferments dietary fiber to produce SCFAs, most notably butyric acid (93). Butyrate is the preferred energy source for colonocytes and has anti-inflammatory effects by inducing Tregs and promoting epithelial barrier permeability (94). SCFAs can contribute to the maturation process of dendritic cells in the bone marrow, leading to mature cells with a reduced ability to instigate Th2 responses in the lungs and to induce IgA production by mucosal B cells (94). High levels of gut microbial-derived butyrate in early life reduce the risk of allergen sensitization and asthma occurrence later in life, both in experimental and human studies (94, 95).

Finally, a recent systematic review of studies examining the effect of oral probiotic supplementation on asthma-related outcomes reported no significant differences in children receiving probiotics compared to the control groups regarding asthma control and lung function (96).

## Conclusion

Early life is a crucial period for microbiome and immune development. The perturbation of the development and maturation of the microbiome during the first few years of life can have a variety of harmful effects on immune health, contributing to determining the development of atopic diseases. Although current understanding of the relationships between early life nutrition, microbiome, and immune system development has significantly increased in recent years, substantial knowledge gaps persist regarding the molecular mechanisms involved. Understanding these mechanisms is of the outermost importance to develop effective prevention strategies for allergic diseases.

## Author Contributions

DP, GN, IT, PC, and MD made substantial contributions to conception, design, and acquisition of data. GN, IT, PC, and MD drafted the initial manuscript. DP, PC, and MD critically reviewed it for important intellectual content. All authors gave the final approval of the version to be published.

## Conflict of Interest

The authors declare that the research was conducted in the absence of any commercial or financial relationships that could be construed as a potential conflict of interest.

## Abbreviations

AD: atopic dermatitis
BM: breast milk
CMA: cow’s milk allergy
FA: food allergy
SCFA: short-chain fatty acid
TLRs: Toll-like receptors
Treg: regulatory T cell.
